# A mixed methods inquiry: How dairy farmers perceive the value(s) of their involvement in an intensive dairy herd health management program

**DOI:** 10.1186/1751-0147-50-50

**Published:** 2008-12-18

**Authors:** Erling Kristensen, Carsten Enevoldsen

**Affiliations:** 1 StrateKo Aps, Gartnervaenget 2, DK-8680 Ry, Denmark; 2 Department of Large Animal Sciences, Faculty of Life Sciences, University of Copenhagen, Grønnegaardsvej 2, DK-1870 Frederiksberg C, Copenhagen, Denmark

## Abstract

**Background:**

Research has been scarce when it comes to the motivational and behavioral sides of farmers' expectations related to dairy herd health management programs. The objectives of this study were to explore farmers' expectations related to participation in a health management program by: 1) identifying important ambitions, goals and subjective well-being among farmers, 2) submitting those data to a quantitative analysis thereby characterizing perspective(s) of value added by health management programs among farmers; and 3) to characterize perceptions of farmers' goals among veterinarians.

**Methods:**

The subject was initially explored by means of literature, interviews and discussions with farmers, herd health management consultants and researchers to provide an understanding (a concourse) of the research entity. The concourse was then broken down into 46 statements. Sixteen Danish dairy farmers and 18 veterinarians associated with one large nationwide veterinary practice were asked to rank the 46 statements that defined the concourse. Next, a principal component analysis was applied to identify correlated statements and thus families of perspectives between respondents. Q-methodology was utilized to represent each of the statements by one row and each respondent by one column in the matrix. A subset of the farmers participated in a series of semi-structured interviews to face validate the concourse and to discuss subjects like animal welfare, veterinarians' competences as experienced by the farmers and time constraints in the farmers' everyday life.

**Results:**

Farmers' views could be described by four families of perspectives: Teamwork, Animal welfare, Knowledge dissemination, and Production. Veterinarians believed that farmers' primary focus was on production and profit, however, farmers' valued teamwork and animal welfare more.

**Conclusion:**

The veterinarians in this study appear to focus too much on financial performance and increased production when compared to most of the participating farmers' expectations. On the other hand veterinarians did not focus enough on the major products, which farmers really wanted to buy, i.e. teamwork and animal welfare. Consequently, disciplines like sociology, economics and marketing may offer new methodological approaches to veterinarians as these disciplines have understood that accounting for individual differences is central to motivate change, i.e. 'know thy customer'.

## Background

More than two decades have passed since Bigras-Poulin and co-authors [1] in a classical paper demonstrated that the farmer's socio-psychological characteristics are more important to farm performance than the herd level variables describing production, health and fertility. The perspective brought forth by Bigras-Poulin *et al*. finds support in other scientific fields like management, rural sociology and economic psychology. These disciplines acknowledge that people take actions for a variety of reasons like relative income standing [2], risk aversion [3], a feeling of uncertainty [4], employee satisfaction [5] and subjective well-being [6]. Nonetheless, research has remained scarce in veterinary science when it comes to the motivational and behavioral side of farmers' perspectives and overall decision utility in relation to disease and health [7], perhaps because it is complex, context-related, and contains elements that cannot be addressed with the research methodologies usually applied in veterinary science?

Studying farmers' expectations and subsequent valuation when participating in a herd health management (HHM) programs requires an interdisciplinary approach [8-11]. This is needed to understand the variables, relationships, dynamics and objectives forming the dairy farm context, e.g. time-dependent variables related to cows and herd(s) as well as variables dealing with the farmer's goals and attitudes.

The distribution of limited resources between herd health and production and between overall farm performance and personal leisure and preferences sums up to a very complex and farm specific equation or context. Choices in this equation reveal preferences and define decision utility. Thus, studying farmers' choices may reveal farmers' expectations from participating in a HHM program. However, farmers' decision making is obviously not confined to herd health, explaining why the level of investment in management systems may not always be the 'optimal' level [12].

The objectives of this study were to study farmers' expectations related to participation in a HHM program by: 1) identifying important ambitions, goals and subjective well-being among farmers, 2) submitting those data to a quantitative analysis thereby characterizing perspective(s) of value added by health management programs among farmers; and 3) to characterize perceptions of farmers' goals among veterinarians.

## Methods

### Q-factor analysis

In this study we needed to address the dairy farmers' subjective points of view and the veterinarians' perception of dairy farmers' points of view. The question was: How do dairy farmers perceive the value(s) of their involvement in an intensive dairy herd health management program?

The core research tool of this study was Q-methodology, which was first described by Stephenson [13] and provides a foundation for the systematic study of subjectivity, that is, 'a person's viewpoint, opinion, beliefs, attitude, and the like' [14]. Consequently, Q-methodology does not aim at estimating proportions of different views held by the 'farmer population' (this would require a survey). Rather, Q identifies qualitative categories of thought shared by groups of respondents, i.e. farmers.

We followed the guidelines described by van Exel and Graaf [15], who divide the approach into the following steps:

1. Construction of the concourse

2. Development of the Q-set

3. Selection of the P-set

4. Q-sorting

5. Q-factor analysis

#### 1. Construction of the concourse

In Q-methodology a 'concourse' refers to 'the flow of communicability surrounding any topic' [14]. The concourse is a technical concept for a contextual structure of all the possible statements that respondents might make about their personal views on the research question. In this study, the concourse was constructed by the authors' reflections on viewpoints in literature, our experience, and previous interviews and discussions with dairy farmers, veterinarians and researchers. This concourse supposedly contains the relevant aspects of all the discourses and thus forms the raw material for Q-methodology.

#### 2. Development of the Q-set

The concourse is subsequently broken down into answers or statements that potentially could answer the research question (Table 1). Next, a subset of statements is drawn from the concourse (labeled the Q-set). The selection may be based on existing hypotheses or theory. The Q-set should include statements that are contextually different from one another in order to ensure a broad representation of points of view in the Q-set [16]. In this study all the 46 statements derived from the concourse were included in the Q-set to keep as broad a representation of points of view as possible.

**Table 1:** The idealized (weighted and normalized) Q-sorting within each family of farmers' perspectives.

	Statements derived from the concourse^1^	Family 1: Team work	Family 2: Animal welfare	Family 3: Knowledge	Family 4: Production
1	I make more money with the management program	2	0	2	0

2	Team spirit increases in the dairy setting	1	-1	-1	3

3	It makes antibiotics more available	-1	4	-1	0

4	It is an insurance of the production level	-1	-1	0	2

5	I like to be 'up front'	-3	-1	4	-1

6	I can outsource the responsibility of herd health	-3	-4	-4	-3

7	It gives the vet a chance to prove his worth	-2	-3	-2	3

8	Future insurance: The vet knows me and the herd	0	0	2	0

9	I want to make a contribution to develop the advisory service	-3	0	0	4

10	Reproduction increases	3	3	0	2

11	I get whole-farm consultancy	1	-2	2	1

12	A high management level in the stable vs. grazing	-1	0	-5	-5

13	It is preferable to the image of dairy industry – and me	-2	2	-3	2

14	Incidence of disease decreases	0	3	-3	-2

15	The vet and I share responsibility regarding herd health	-2	-1	-3	0

16	The vet updates me on the newest knowledge	-1	1	5	-2

17	More cows can be treated without paying the vet	-2	4	-2	-5

18	I like that only one vet works with me and my herd	1	3	1	4

19	Yield increases	4	1	-1	1

20	I work more systematically, when someone checks up on me	0	-2	0	-4

21	The vet has more experience than me	0	0	1	0

22	My understanding of herd dynamics as a whole increases	-1	-1	3	1

23	The vet and I work better together	4	2	1	3

24	My financial lenders requested it	-5	-5	-4	-1

25	The vet made me an offer I could not refuse	-5	-2	-5	-2

26	It is necessary for me to take in the herd size	2	-3	3	0

27	Incidence of mastitis decreases	0	3	-3	-1

28	Nothing is missed – and it increases joy in my work life	1	1	-2	-2

29	I need a loyal and independent advisor to spar with	1	-1	4	-4

30	It enhances the business aspect of my herd	-4	-3	3	-1

31	It was recommended to me (by farmers, consultant)	-4	-5	-4	-3

32	Incidence of dead animals decreases	2	-4	-2	-4

33	The vet said it was a good idea	-3	2	3	-1

34	It is a current trend – and I like new ideas	-4	-3	5	-1

35	The vet helps to educate my staff	-1	-2	-2	1

36	The vet bill decreases in the long run	0	1	2	-3

37	It gives me an opportunity to evaluate the effect of interventions	1	2	2	2

38	My knowledge on cows and herd increases	3	-2	-1	5

39	The vet is more enthusiastic regarding my problems	4	-4	0	-2

40	The vet helps to put up relevant performance indicators	3	0	4	1

41	I prefer prevention to treatment	5	5	0	4

42	The vet gets deep insight into the herd – better advices	5	1	1	2

43	I can exploit the vets knowledge more systematically	3	2	1	3

44	Time is saved due to systematic work procedures	2	1	-1	5

45	Animal welfare and herd health increases	2	4	1	1

46	Extended HHM programs reduce the use of antibiotics	-2	5	-1	-3

% variance attributable to each family of farmers' perspectives (unrotated factors(rotated factors))		37/22	12/18	9/13	7/12

^1 ^A concourse is a 'view of the world' constructed by the researcher from various sources of data. In Q-methodology the concourse is broken down by the researcher into a number of statements that respondents rank according to 'my point of view', i.e. how well the individual statement presents an answer to the research question

i.e. how a hypothetical respondent with a 100% loading on that factor would rank all the statements according to the guide for ranking

#### 3. Selection of the P-set

The P-set is a sample of respondents, which is theoretically relevant to the research question, i.e. it represents persons who probably will have clear and distinct viewpoints on the subject and, because of that quality, may define a factor [15]. Sixteen farmers were selected from a group of Danish dairy farmers managing conventional dairy enterprises and being clients in a single large nationwide cattle practice and participating in a recently developed intensive HHM program. Farmers were selected that we expected would provide breath and comprehensiveness to the P-set (Table 2) thereby acknowledging that the P-set is not supposed to be random [17]. The selected farmers (the P-set) were invited to participate in the study by a covering letter, an additional page describing the 'conditions of instruction' [14], an empty layout guide and a stamped envelope for the returning of the layout guide. Farmers did not receive any compensation for their participation.

**Table 2:** Summary of characteristics of the herds of the farmers participating in the semi-structured interviews

Characteristics	1	2	3	4	5	6	7	8	9	10	11
Cows per year^1^, n	105	140	115	123	161	141	106	137	92	141	182

ECM per cow per year, kg	8,908	9,932	8,276	7,943	9,847	9,420	8,898	10,050	10,712	10,023	9,722

Age at 1st calving, Months	25,3	25,4	28,7	26,0	27,9	25,9	25,7	25,7	25,5	26,3	24,9

Culling-rate^2^	30	48	37	73	34	30	38	40	36	59	52

Bulk tank somatic cell count, 1000 per ml	220	216	385	299	323	235	224	201	227	403	186

Milk delivered, percent of produced^3^	95	98	98	92	96	91	98	92	90	95	91

Automatic Milking System	No	No	No	Yes	No	No	No	No	No	Yes	No

Age of farmer, intervals	> 50	> 50	40–50	40–50	> 50	> 40	40–50	40–50	> 50	< 40	< 40

^1 ^Cows per year = total number of cow days in a year/365

^2 ^Calculated according to the Danish definition: (number of cows going into the herd plus number of cows leaving the herd)/2/number of cows per year

^3 ^Percentage of milk shipped to the dairy of milk produced

#### 4. Q-sorting

Respondents (P-set) were asked to rank (Q-sort) the statements (Q-set) according to their own point of view with minimum interference from our part. The fact, that the farmers ranked the statements from their own point of view and not according to 'facts', is what brings the subjectivity into the study. The statements were sorted on the layout guide along a quasi-normal distribution (mean 0, SD 2.67) ranging from 'agree mostly' (+5) to 'disagree mostly' (-5). Each of the statements was typed on a separate card and marked with a random number for identification.

During a continuing education course in November 2007, 18 experienced veterinarians associated with the abovementioned cattle practice sorted the same statements in a similar manner as the farmers. Here, the 'conditions of instructions' were delivered in a short oral presentation.

#### 5. Q-factor analysis

The returned Q-sortings from the farmers and veterinarians were analyzed separately by means of the PC-program 'PQMethod' [18] that is tailored to the requirements of Q-methodology. Specifically, 'PQMethod' allows easy entering of data the way it was obtained, i.e. as 'piles' of statement numbers. 'PQMethod' computes correlations among the respondents (the variables or columns in the data matrix) that were characterized by the Q-sorting. That is, each of the 46 statements was represented by one row in the matrix. This is equivalent to reversing the correlation matrix used in traditional 'R-factor analysis', which is based on correlations between variables characterizing respondents. Respondents, who are highly correlated with respect to their ranking of statements, are considered to have a 'familiar' resemblance, i.e. those statements belonging to one family being less correlated with statements of other families. A principal component analysis was chosen in 'PQMethod' to estimate the total explained variance and the variance attributable to each identified factor (family of perspective). Following a commonly applied rule for including number of factors, factors with eigenvalues smaller than 1.00 were disregarded. A factor loading was determined for each respondent as an expression of which respondents were associated with each factor and to what degree. Loadings are correlation coefficients between respondents and factors. The remaining factors were subjected to a varimax (orthogonal) rotation to provide the rotated factor loadings (Table 3).

**Table 3:** Rotated factor loadings of each of the participating farmers on the selected factors where 'X' indicates a defining sort (*P *< 0.05)

Farmer	Factor 1	Factor 2	Factor 3	Factor 4
1	0.12	-0.10	0.87X	0.00

2	0.70X	0.15	0.32	-0.24

3	0.72X	0.43	-0.07	0.02

4	0.12	0.86X	-0.02	0.22

5	0.66X	0.27	0.09	0.37

6	-0.02	0.40	0.60X	0.19

7	0.25	0.80X	0.06	-0.22

8	0.57	0.27	0.48	0.25

9	0.49	-0.29	0.14	0.56

10	0.36	0.30	0.41	0.44

11	0.08	0.49	0.22	0.46

12	0.65X	0.07	0.08	0.25

13	0.13	0.65X	0.40	0.19

14	0.18	0.14	0.05	0.79X

15	0.76X	-0.05	-0.02	0.23

16	0.55X	0.22	0.43	0.16

The final step before describing and interpreting the factors was the estimation of factor scores and difference scores. A statement's factor score is the normalized weighted average statement score of respondents that define that factor. The weight (w) is based on the respondent's factor loading (f) and is calculated as: w = f/(1-f^2^). The weighted average statement score is then normalized (with a mean of 0.00 and SD = 1.00) to remove the effect of differences in number of defining respondents per factor thereby making the statements' factor scores comparable across factors. Thus, we take into account that some respondents are closer associated with the factor than others by constructing an idealized Q-sorting for each factor. The idealized Q-sorting of a factor may consequently be viewed as how a hypothetical respondent with a 100% loading on that factor would have ranked all the statements on the layout guide. The idealized layout guides for each family of farmers' perspectives are provided in Table 1. The difference score is the magnitude of difference between a statement's score on any two factors that is required for it to be statistically significant. 'PQMethod' offers the possibility to identify the most distinguishing statements for each family of perspectives, i.e. when a respondent's factor loading exceeds a certain limit (often based on *P *< 0.05) and consensus statements between the families of perspectives, i.e. those that do not distinguish between any pair of families [15]. The limit for statistical significance of a factor loading is calculated as: Factor loading/(1 divided by the square root of the number of statements in the Q-set) [15]. If this ratio exceeds 1.96, the loading was regarded as statistically significant (*P *< 0.05). The idealized Q-sortings were assigned with informative names (labels) with input from both the most distinguishing statements for family of perspective and the consensus statements. The process of giving names to the idealized Q-sortings according to its characteristics may serve to facilitate the discussion and communication of the findings [19].

### The semi-structured interviews

All farmers in the P-set were invited to participate in an interview to elaborate on their preferences as expressed by the placing of the statements on the layout guide and 12 farmers accepted the invitation. All farmers were men and managed conventional farms, all free-stalls. Additional herd characteristics are listed in Table 2. Veterinarians were not interviewed due to budget and time constraints. The first farmer accepting the invitation was defined to serve as a pre-test for the interview approach (leading to minor adjustments). This interview was eliminated from the data. The qualitative study therefore consisted of 11 interviews. Consequently, the entire data collection process was as follows: First, veterinarians face-validated the contextual structure of the concourse during the common Q-sorting session. Second, pre-testing was performed. Third, farmers sorted the Q-set and returned the layout guides. Fourth, the contextual structure of the concourse and the results from the individual Q-sortings were face-validated by the farmers during the interviews. Further, the interviews offered an opportunity to confirm farmers' understanding of the sorting technique and correct any misunderstandings. No misunderstandings were identified. Fifth, following the face-validation of the concourse each interview session with the 11 farmers included three thematic questions:

• What about animal welfare and herd health?

• Assume that you have an extra hour every day (i.e. the 25th hour) what would you do? – Increase the herd size, improve management or increase leisure time?

• Assuming you have a farm board: Would your practicing veterinarian be a member? – why/why not?

The interviews followed the approach described by Vaarst *et al*. [9] and lasted between 65 and 80 minutes. Interviews were digitally recorded and all interviews were administered (January to March, 2008) by the first author. The interviews were analyzed according to the inductive approach discussed by Kristensen *et al*. [8] for HHM research with inspiration from [20] on how to interpret a series of interviews with the intent to provide insight into a phenomenon of more general interest, e.g. to facilitate 'multivoices' [21].

## Results

### Q-factor analysis

The concourse was a primary result. Essentially, both farmers and veterinarians accepted the concourse by face-validation, i.e. farmers before the interview sessions and veterinarians before and during the sorting process. Four families of farmers' perspectives (idealized Q-sorts) were identified with the Q-factor analysis. They explained a total of 65% of the variance between farmers. Table 4 illustrates the most distinguishing statements (*P *< 0.05) for each family of perspectives. Consensus statements (non-significant at *P *> 0.05) were: 1, 2, 4, 6, 8, 10, 15, 18, 21, 23, 31, 35, 37, 43, and 45. These statements were considered equally revelatory by virtue of their salience, i.e. none of the farmers placed much value on these statements be it positive or negative value.

**Table 4:** The most distinguishing statements (*P *< 0.05) for each family of farmers' perspectives in decreasing order by idealized factor scores^1^, respectively

Family most distinguishing statements	No. 1	No. 2	No. 3	No. 4	No. 5	No. 6	No. 7	No. 8
Family 1: Teamwork	39^2^	32^2^	14	9^2^	-	-	-	-

Family 2: Animal welfare	46^2^	17^2^	45	3^2^	14	27	38	26

Family 3: Knowledge dissemination	34^2^	16^2^	29^2^	5^2^	30	41	38	44

Family 4: Production	44	9^2^	7^2^	24	36	29^2^	17	-

^1 ^The idealized Q-sorting of a factor may be viewed as how a hypothetical respondent with a 100% loading on that factor would have ranked all the statements on the layout guide

^2 ^*P *< 0.01

Ranking of statements by idealized factor scores combined with the insight obtained from both the most distinguishing statements and the consensus statements were submitted to a qualitative analysis with the insight obtained by the first author during the series of interviews into the farmers' lived experiences, perspectives and expectations. The purpose of this analysis was to construct informative names (labels) to each identified family of farmers' perspectives. The selected names to describe families of farmers' perspectives were (in decreasing order by explained variance, see Table 1):

• Teamwork

• Animal welfare

• Knowledge dissemination

• Production

Equally, four families of veterinarians' beliefs on farmers' perspectives were identified explaining a total of 69% of variance. Informative names were identified by means of a qualitative analysis of the results, i.e. combining the idealized Q-sorts and the five most preferred statements from each family of veterinarians' perception of farmers' perspectives (not shown). It was realized that the family names from the farmers' families of perspectives could be re-used as 'PQMethod' identified a number of veterinarians' families of perspectives equal to the families of farmers' perspectives. The families of veterinarians' perception of farmers' perspectives explained 48%, 9%, 6% and 6% of variance for families Production, Animal welfare, Knowledge dissemination and Teamwork, respectively.

### The semi-structured interviews

The raised question regarding animal welfare and herd health (AWHH) divided farmers into two points of view. Farmers associated with the first viewpoint explained their interest in AWHH primarily as a consequence of society's scepticism towards the production system of dairy industry as experienced by the farmers, i.e. *'people are watching us' *and *'society thinks, that farmers are the kind of people that beat up animals'*. Farmers sharing the second viewpoint believed that HHM was an important tool to increase AWHH. These farmers explained that an increase of AWHH was an inevitable consequence of the HHM program. However, the follow-up question: *'Why do you value AWHH' *revealed that farmers associated with the second viewpoint had to be divided into two sub-views to be meaningfully described. The farmers belonging to the first sub-viewpoint placed value on AWHH because of the farmers' firm belief that AWHH is a precondition to increase the overall farm production, i.e. *'I tell you, animal welfare and economy is really closely connected. The reason that I care about animal welfare is because it is a financially reasonable way to do things' *and *'it's obvious that we are quite interested in increasing animal welfare because it will improve the financial bottom-line in the long run'*. Farmers sharing the second sub-viewpoint experienced AWHH to hold a unique value associated with their subjective well-being. These farmers emphasized a feeling of personal satisfaction related to being around healthy animals, providing the farmers with a feeling of 'a job well done', i.e. *'animal welfare reflects other values in our lives' *and *'I have a philosophy on animal welfare; the day I can't tend to each cow as well as the time I had twenty, then I have too many cows'*. Farmers from both sub-viewpoints stated (even though it was not a specific question) that AWHH and the cost of the HHM program had to compete for limited resources (primarily time and money) with other investment opportunities (e.g. the dairy business, the farmer's subjective well-being related to values provided by the HHM program, family) both on and off the farm in terms of expected return on investment.

The second thematic question related to farmers' time-budget. We suggested that each farmer was given an extra hour every day, i.e. the 25th hour. Farmers were divided into four points of view based on their different viewpoint on how to spend this extra time: 1) Farmers associated with the first viewpoint wanted to increase leisure time. The explanations were primarily found within two subjects: Family; *'it is really important to me that I am a visible dad'*; Daily stress: *'I constantly feel that my presence is needed; therefore I have an unsatisfied need to experience freedom'*; 2) The second viewpoint included farmers that clearly stated they would choose to increase management within the present framework of the dairy farm, i.e. *'I would try to correct the errors that I do not have the time to at the moment' *and *'one extra hour is not enough at all. There are so many things in my daily work that I could improve – but I do not have the time'*. Some of the farmers related to the second viewpoint elaborated on the question and explained that they would have liked to answer 'family', however, realities were likely to be different, i.e. *'looking at myself, I sometimes feel that I should have spent more time with my family, you know, gone with the kids to soccer, but I also know that if this 25th hour was really true, I would probably not follow the kids, but go into stable and try to improve something – even though it really wasn't, what I wanted to do'*; 3) Farmers from the third viewpoint asked if it was an acceptable answer to increase management with the intent to provide a basis for a near-future expansion of the herd size; 4) Last, farmers sharing the fourth viewpoint stated that given extra time they would buy more cows *'because an increasing number of cows leads to an increasing number of employees, making it possible to run the farm without my daily presence'*. From all of the abovementioned viewpoints a common viewpoint could be summarized: It is necessary that veterinarians include opportunity time in addition to a strict focus on profitability (and welfare?) when proposing recommendations.

It was the farmers' experience that veterinarians knew almost nothing about herd health economics, finances in general or strategy related to running a business. However, the farmers expressed a willingness to buy such a service if provided by a veterinarian able to combining the classical veterinary disciplines with management, strategy and finances.

## Discussion

### Validity of results

The objective of this study was not to generalize possible findings to the whole population of farmers or veterinarians but to obtain insight into a phenomenon as experienced by a range of individuals selected for this study because of their 'information richness' [22]. Consequently, results are only directly applicable to the particular participants, settings and contexts [23]. However, the active participation of the end-users, i.e. farmers and veterinarians, in the modelling-validating process is emphasized as an important part of the usefulness dimension of validity in operations research [24]. Further, we have taken into consideration the length of the interviews and the number of interviewees to increase the likelihood of data saturation as discussed by Onwuegbuzie and Leech [23]. These authors studied literature and have presented a sample size guideline to qualitative research. In phenomenological research 6–10 interviewees are recommended when homogeneous samples are selected for interviews. We regard our sample as homogenous because all the participating farmers are associated with the same veterinary practice and have chosen to be involved in the same intensive HHM program. Additionally, Onwuegbuzie and Leech [23] present their reflections regarding the importance of the length of each contact to reach informational redundancy. The length of our interviews followed the description by both Vaarst *et al*. [9] and Onwuegbuzie and Leech [23]. Morse [25] defines the concept of 'saturation' in qualitative data as 'data adequacy' and adds that it is 'operationalized as collecting data until no new information is obtained'. Consequently, the face-validation of the concourse by farmers and veterinarians may be seen as an acceptance of a 'saturation' of perceptions of the Q-set providing the data with 'interpretive sufficiency' to take into account the multiple interpretations of life [26].

Q-Methodology is about respondents ranking matters of opinion within a concourse to identify the existence of families of perspectives. Consequently, the results of a Q-factor analysis is useful to identify and describe a population of viewpoints and not, as in R, a population of people [27]. The difference between Q and R being that the issue of large numbers, so fundamental to R, becomes rather unimportant in Q [16]. The most important type of reliability for Q is replicability: Will the same 'condition of instruction' lead to factors that are schematically reliable, that is, represent similar families of perspectives on the topic? [15]. In contrast to most studies, Q-studies cannot obtain 'true replication' because: 1) an identical set of participants, contexts and experiences is impossible to find and; 2) the concourse as it expresses itself in a Q-study becomes context-bound to the particular participants, settings and contexts. It follows that the present Q-study could not be replicated with the same farmers as participants because these farmers were likely to have reflected on the Q-sorting and the interviews making them 'different persons' than in the beginning of the study. Thomas and Baas [28] concluded that scepticism related to the issue of reliability is unwarranted as the objective in Q-studies is to reach an in-depth understanding of the context in question and thus requires an equally in-depth understanding of a different context to draw possible inferences between the two different contexts. The results of a Q-study are the distinct families of perspectives on a topic (as described by the concourse) that are operant, not the percentage of the sample (or the general population) that adheres to any of them. This would require a (questionnaire) study of a representative sample of people and such a study could be relevant as a follow-up to this study. 'Quality is operationally distinct from quantity' [16]. Consequently, the required number of respondents to establish the existence of a factor is substantially reduced for the purpose of comparing one factor with another compared to traditional R statistics [15].

### General discussion

In this study farmers' statements could meaningfully be placed into four groups with distinctly identified differences related to the individual farmers' perception of value added by a HHM program. Maybery and co-authors [29] applied a different technique but reported analogous findings in a study on economic instruments and common good interventions in Australia. Kiernan and Heinrichs [19] discussed how information on similarities between groups of farmers may be utilized by veterinarians to increase the effectiveness of management programs.

The Q-factor analysis divided farmers' perspective on HHM programs labeled as: Teamwork, Animal welfare, Knowledge dissemination and Production, respectively. Veterinarians believed the correct order to be: Production; Animal welfare; Knowledge dissemination and Teamwork, respectively. It follows that the veterinarians' perception of farmers' perspective as compared to the farmers' expectations were quite different. From the explained variances it follows that most farmers are correlated with Teamwork and most veterinarians are correlated with Production. Potentially, this difference may lead to differences of opinion when the farmer and veterinarian, respectively, evaluate the impact or success of a HHM program. The veterinarian believes that the success criterion is increased production and subsequent profit whereas the farmer expects to be part of a team working with shared ambitions and common goals.

Farmers focusing on AWHH were divided between those focusing on an expected correlation between increases in AWHH and financial performance and those focusing on a feeling of increased subjective well-being from being around healthy cows. This is an important finding, which is also discussed in details by Kristensen *et al*. [30] illustrating how 'qualitative studies can be added to quantitative ones to gain better understanding of the meaning and implications of the findings' [31].

This study has provided evidence that it is unlikely that (all) the time saved due to systematic work procedures implemented by a HHM program is re-invested in production to increase financial performance. Obviously, the potential increase in financial performance is not realized if time is allocated towards leisure and away from production. Trying to understand and predict human behaviour primarily on monetary incentives is problematic [2,32] as income only explains about 2–5% of the variance related to measures of subjective well-being [6]. Further, farmers' decision making obviously is not confined to herd health [33]. In practice, the level of investment in management systems will never be the 'optimal' solution from a herd health perspective, because 1) investment prospects are better elsewhere [12]; 2) value added to overall financial performance is measured by a different currency than money [7]; and 3) short-term gains are valued more than a possible larger future gain predicted by a model or a HHM program [6].

A marked discrepancy was identified between the family of veterinarians that focused on production and how farmers view the veterinarians' competences in areas like business, farm management etc. Most veterinarians correlated with production; however, none of the farmers would ask their veterinarian to sit in a farm board because of what the farmers perceived as a general lack of knowledge on farm management and a more specific lack of knowledge on strategy and finances. De Kruif and Opsomer [34] report similar findings. The farmers, however, expressed an interest in buying such a service if provided by an experienced veterinarian able of combining the classic veterinary disciplines with the disciplines of business and management. The overall impression from the interviews was that farmers view their affiliated veterinarian as a 'master' of the classical veterinary virtues (diagnostics and treatment at cow-level and to some extent herd-level) but much less qualified to handle the management aspects of HHM consultancy. This finding may be important to veterinary schools, as changes in the educational structure towards 'whole farm' management seem warranted.

### Implications of results to the herd health management community

To date, most research on subjective well-being has focused on the well-being of the individual, i.e. the farmer [35]. This study suggests that there may be good reasons to draw veterinarians' attention to the overall well-being of the farmer's household.

*Where to go from here? *If different farmers are motivated by very different factors then a stereotype 'one-size-fits-all' approach from veterinarians to stimulate improvements of management obviously is unlikely to succeed. The veterinarians in this study appear to focus too much on financial performance and increased production when compared to farmers' expectations. On the other hand veterinarians apparently did not focus enough on a major product, which farmers really wanted to buy, i.e. teamwork and whole farm management. Consequently, disciplines like sociology, economics and marketing may offer new methodological approaches to scientists and veterinarians as these disciplines have long been based on the understanding that accounting for individual differences is central to understand the stimulus for change, i.e. 'know thy customer' [29].

## Conclusion

Farmers' expectations related to a HHM program could be divided into four families: Teamwork was most important followed by Animal welfare, Knowledge dissemination, and Production. Animal welfare was highly valued by farmers, but for varying reasons. In contrast, the dominant view of veterinarians was that farmers focused mainly on production and financial performance and least on the value of teamwork. Farmers, however, perceived veterinarians as largely incompetent in areas like finances and business management and would not invite their veterinarian to be a member of their farm board. These differences of perspectives and thus expectations to value added by a management program between farmers and veterinarians have implications for the future herd health management research and education. If dairy farmers value teamwork more than production and profit, as indicated by this study, veterinarians would be wise to change their focus or increase their abilities in combining veterinary science with knowledge on management and finances as this service was requested by, but apparently not available to, the dairy farmers. Equally, changes in pre-graduate veterinary education directed towards 'whole farm' management seem warranted.

## Abbreviations

HHM: Herd health management; AWHH: Animal welfare and herd health.

## Competing interests

The authors declare that they have no competing interests.

## Authors' contributions

EK collected and analysed the data, did the literature review and drafted the manuscript. CE made substantial contributions to the conceptual ideas and revision of the manuscript for important intellectual content in detail. Both authors read and approved the final manuscript.
